# RANK–RANKL–OPG Axis in MASLD: Current Evidence Linking Bone and Liver Diseases and Future Perspectives

**DOI:** 10.3390/ijms25179193

**Published:** 2024-08-24

**Authors:** Federico Monti, Federica Perazza, Laura Leoni, Bernardo Stefanini, Silvia Ferri, Francesco Tovoli, Guido Zavatta, Fabio Piscaglia, Maria Letizia Petroni, Federico Ravaioli

**Affiliations:** 1 Department of Medical and Surgical Sciences, IRCCS Azienda Ospedaliero-Universitaria di Bologna, 40138 Bologna, Italy; federico.monti15@studio.unibo.it (F.M.); federica.perazza@studio.unibo.it (F.P.); bernardo.stefanini@unibo.it (B.S.); francesco.tovoli@unibo.it (F.T.); guido.zavatta@unibo.it (G.Z.); fabio.piscaglia@unibo.it (F.P.); marialetizia.petroni@unibo.it (M.L.P.); 2 Department of Dietetics and Clinical Nutrition, Maggiore-Bellaria Hospital, Azienda Unità Sanitaria Locale (AUSL), 40138 Bologna, Italy; leoni_laura@hotmail.it; 3 Division of Internal Medicine, Hepatobiliary and Immunoallergic Diseases, IRCCS Azienda Ospedaliero-Universitaria di Bologna, 40138 Bologna, Italy; silvia.ferri@aosp.bo.it; 4 Division of Endocrinology and Diabetes Prevention and Care, IRCCS Azienda Ospedaliero-Universitaria di Bologna, 40138 Bologna, Italy; 5 Unit of Clinical Nutrition and Metabolism, IRCCS Azienda Ospedaliero-Universitaria di Bologna, 40138 Bologna, Italy

**Keywords:** bone metabolism, metabolic dysfunction-associated steatotic liver disease, metabolic-associated steatohepatitis, chronic liver disease, osteoporosis, osteopenia, vitamin D

## Abstract

Metabolic dysfunction-associated steatotic liver disease (MASLD)—and its worse form, metabolic-associated steatohepatitis (MASH), characterised by inflammation and liver damage—corresponds to the liver’s involvement in metabolic syndrome, which constitutes an economic burden for healthcare systems. However, the biomolecular pathways that contribute to steatotic liver disease are not completely clear. Abnormalities of bone metabolism are frequent in people affected by metabolic liver disease, with reduced bone density and an increased risk of fracture. Receptor activator of NF-κB (RANK), receptor activator of NF-κB ligand (RANKL), and osteoprotegerin(OPG) are critical regulators of bone metabolism, performing pleiotropic effects, and may have potential involvement in metabolic disorders like MASLD, resulting in a topic of great interest and intrigue. This narrative review aims to investigate this potential role and its implications in MASLD development and progression and in hepatocellular carcinoma, which represents its worst complication.

## 1. Introduction

Metabolic dysfunction-associated steatotic liver disease (MASLD) [1], previously named non-alcoholic fatty liver disease (NAFLD), is the most common liver disease, and it affects about 25% of the world adult population [2]. The increase in its incidence and prevalence is connected to the global rise in overweight and obesity [3]. 

MASLD is associated not only with obesity, but also with multiple and severe comorbidities, such as cardiovascular disease and type 2 diabetes (T2D). MASLD and T2D present a strong correlation, probably due to the important role of insulin resistance in the onset of both conditions [4]. Furthermore, they share a common relevant comorbidity, namely, osteoporosis. 

Osteoporosis is a bone metabolism disorder in which bone becomes more fragile due to mineral density reduction and bone microarchitecture alterations [5]. These abnormalities in skeletal structure lead to an increased risk of fractures. Osteoporosis has been recognised as a frequent complication of MASLD, and recent evidence clearly established their correlation [6]. 

Although MASLD represents a major issue in public health, its pathological mechanisms are not fully understood. Recently, the hypothesis emerged that the link between bone and liver disorders could involve a reciprocal interaction [7]. Multiple studies have investigated the possible involvement—also in MASLD pathophysiology—of molecular mechanisms initially identified as exclusive of bone disease, with a focus on receptor activator of NF-κB (RANK), receptor activator of NF-κB ligand (RANKL), and osteoprotegerin (OPG). 

These belong to the tumour necrosis factor superfamily and are master regulators of bone metabolism [8]. In addition to their critical relevance in bone tissue physiology and pathology, they have been demonstrated to be involved in multiple extraskeletal functions, like immune system development, atherosclerosis, and, recently, metabolic pathways.

In this review, we aim to collect and summarise the current evidence about the possible involvement of RANK, RANKL, and OPG in MASLD pathophysiology, highlighting the potential therapeutic implications that emerge from the studies. Exploring the RANK–RANKL–OPG axis in MASLD enhances our understanding of the disease and opens up promising avenues for therapeutic interventions across hepatology, endocrinology, and bone metabolism.

## 2. Data Sources and Searches

We searched English-language publications in MEDLINE, the Cochrane Library, EMBASE, and PubMed until June 2024. Literature searches were performed using the following keywords: non-alcoholic fatty liver disease, NAFLD, metabolic-associated steatotic liver disease, MASLD, non-alcoholic steatohepatitis, metabolic-associated steatohepatitis, MASH, liver disease, receptor activator of NF-κB, RANK, receptor activator of NF-κB ligand, RANKL, osteoprotegerin, OPG, hepatocellular carcinoma, HCC, insulin resistance, and denosumab.

## 3. RANK–RANKL–OPG Axis in Bone

Contrary to its “static” appearance, bone tissue is extremely dynamic from a metabolic point of view. Bone mass results from continuous and simultaneous bone tissue formation and resorption processes. The main actors in bone metabolism are osteoblasts and osteoclasts, deputed, respectively, to create and destroy bone tissue [9]. While osteoblasts derive from mesenchymal stem cells, osteoclasts consist of multinucleated cells derived from the monocyte–macrophage lineage, deputed to resorbing the mineral matrix of bone tissue [10]. The RANK–RANKL–OPG axis (RROa) emerges as a crucial player in bone metabolism, orchestrating the balance between the activity of osteoblasts and osteoclasts [8].

### 3.1. Receptor Activator of NF-κB (RANK) and Receptor Activator of NF-κB Ligand (RANKL)

The receptor activator of NF-κB ligand (RANKL) is a tumour necrosis factor family cytokine produced by osteoblasts [11] and encoded by the Tnfsf11 gene [12]. Its target consists of the receptor activator of NF-κB (RANK) [13,14], first identified in the late 1980s as a new member of the TNF-receptor family expressed on the surface of osteoclast precursors [15]. 

The interaction between RANKL and RANK, in the presence of macrophage colony-stimulating factor (M-CSF), activates NF-κB and c-jun N-terminal protein kinase (JNK) pathways, stimulating bone resorption through osteoclast differentiation and activation [13,16].

RANKL is synthesised as a trans-membrane protein that can be cleaved into a soluble form by the action of certain proteases [17], probably metalloproteases, that have not been identified yet [18]. Many studies have proved that cell-to-cell interactions are essential for osteoclast differentiation and activation, highlighting the superiority of the membrane-bound form of RANKL [18,19]. This assumption is sustained by several in vitro and in vivo studies conducted in murine models, which demonstrated that soluble RANKL, like other members of the TNF family, is unnecessary for normal skeletal development and is probably unnecessary for its other physiological functions [12,20,21].

### 3.2. Osteoprotegerin (OPG)

Osteoprotegerin (OPG; i.e., to protect the bone) is a soluble glycoprotein, a member of the TNF superfamily that acts as a decoy receptor for RANKL, inhibiting its interaction with RANK [22]. In this way, OPG prevents osteoclast activity and limits bone resorption. This is why, during the initial studies about RROa, OPG was referred to as the osteoclastogenesis inhibitory factor [15], and in vivo and in vitro studies demonstrated that OPG prevents the ultimate stages of osteoclast differentiation without inhibiting osteoclast precursor proliferation [22].

OPG, similar to RANKL, is produced by osteoblasts; so, it is evident that these cells represent the master regulators of bone remodelling, managing the delicate balance between bone reabsorption and production through the preferential expression of RANKL or OPG [8,11]. Intriguingly, Tsukasaki et al. have suggested that different subpopulations of osteoblasts/osteocytes could synthesise RANKL and OPGs [21].

**Figure 1:**
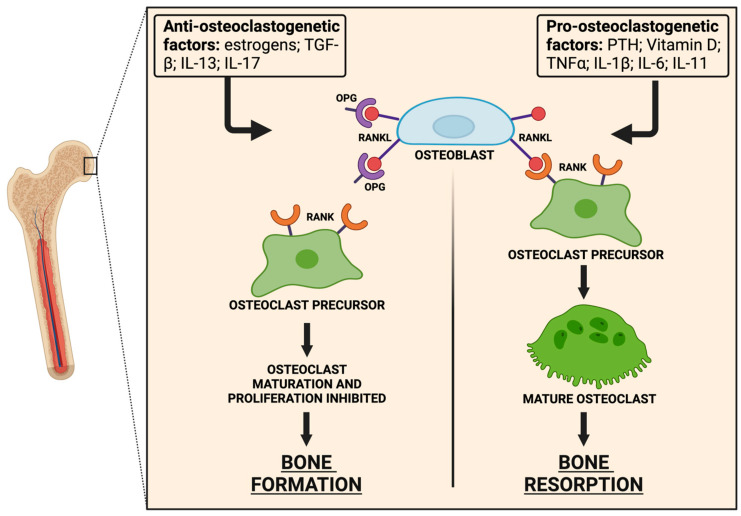
RANK–RANKL–OPG signalling in bone tissue. RANKL–RANK interaction stimulates osteoclast differentiation and activation, promoting bone resorption. On the contrary, OPG improves bone formation preventing RANKL from binding RANK. (Abbreviation: RANKL: receptor activator of NF-κB ligand; RANK: receptor activator of NF-κB; OPG: osteoprotegerin; PTH: parathyroid hormone; TGF-β: transforming growth factor-β; TNFα: tumour necrosis factor-α; IL-1β: interleukin-1β; IL-6: interleukin-6; IL-11: interleukin-11; IL-13: interleukin-13; IL-17: interleukin-17).

Selective expression of OPG rather than RANKL is also influenced by the cytokinic microenvironment and hormones (Figure 1) [23]. Pro-osteoclastogenic factors, like parathyroid hormone, cholecalciferol, tumour necrosis factor α, interleukin-1 β, interleukin-6, and interleukin-11 increase membrane-bound and soluble RANKL production while inhibiting OPG expression [24,25,26]. On the contrary, transforming growth factor β1(TGF-β1) and interleukin-13 reduce RANKL levels and promote OPG production [18].

Post-menopausal status is one of the main risk factors for osteoporosis because oestrogens play a strong anti-osteoclastogenic role [27]. This effect is due to oestrogen’s direct and indirect actions on RROa. In fact, oestrogens stimulate OPG production while inducing osteoclast apoptosis through the reduction of pro-inflammatory cytokines and the resulting deficiency of RANKL [28].

In the close cross-talk between osteoblast and osteoclast, sphingolipid signalling is an important element, favouring the balance of bone metabolism [29].

Lastly, sclerostin, a glycoprotein secreted by osteoblasts and osteocytes, also influences the RROa in the bone tissue. Sclerostin promotes RANKL expression while reducing OPG production, thus inhibiting osteoblastogenesis and stimulating osteoclastogenesis at the same time [30], representing a further mechanism of RRO axis control.

## 4. RROa Studies in Murine Models

Most evidence about RROa physiology comes from studies conducted in murine models, especially in knock-out mice. These protocols allowed for an understanding of the opposite effects of RANKL and OPG action in bone. RANKL and RANK knock-out mice models show generalised and severe osteopetrosis due to impaired osteoclastogenesis [15,31]. On the opposite side, OPG-deficient models present early and severe osteoporosis associated with skeleton abnormalities and precocious onset of fractures that can already occur in the first weeks of life [32,33]. This is due to the significantly augmented osteoclastogenesis and resultant bone reabsorption. 

Knockout murine models have also paved the way to understanding the wide range of extraskeletal functions of RROa, although several are still largely unknown. 

Since RROa are members of the TNF superfamily, it was immediately hypothesised that they should play a part in regulating the immune system’s functions. Indeed, it has been established that RANKL/RANK signalling has a critical role in B- and T-cell maturation [15,31]. For example, it was proved that RANKL-knockout mice present a smaller thymus with significantly reduced cellularity and arrest in the early stages of thymocyte differentiation [31]. 

The most intriguing evidence from both RANK and RANKL knock-out models is the complete absence of lymph node organogenesis but preservation of Peyer’s plaques and other mucosa-associated lymphoid tissues. Recently, Nagashima et al. have demonstrated the importance of RANKL in maintaining the balance between intestinal immune cells and the microbiota [34]. Anderson et al. have demonstrated RANK expression on the surface of dendritic cells induced by CD40L. This evidence intriguingly suggests a possible role of RANK/RANKL in mediating T-cell and dendritic cell interaction [14], which has also been proposed in murine models with RANKL gene deletion [31]. The bone tissue and the immune system share a common embryological origin, and the dual role of RANK–RANKL in both bone metabolism and immune system regulation is probably due to the concurrent development of the skeletal system and specific immunity during vertebrate evolution [35]. The main and most unexpected finding from murine models with complete deletion of OPG was the increase in vascular calcifications detected in the aorta and other big arteries [32]. Many observational studies found an association between post-menopausal osteoporosis and atherosclerosis [36,37]. However, the research in OPG knock-out mice suggests that OPG could be the link between the bone and arteries, playing a part in the pathophysiology of degenerative arterial disease. It must be noted that conflicting results have emerged from animal and human studies about the possible role of OPG [38].

## 5. RROa in MASLD

In recent years, the pleiotropic effects of RROa have emerged thanks to the evidence that RANK, RANKL, and OPG are widely expressed by multiple cell types, such as adipocytes, hepatocytes, and even pancreatic beta cells [39,40,41,42]. As a result, researchers began to investigate the possible role of RROa in metabolic pathways and, consequently, in dysmetabolic disorders, including metabolic dysfunction-associated steatotic liver disease (MASLD).

The effects of RROa in MASLD are widely discussed due to the need for more specific studies and the profound heterogeneity of the results. In a study of 82 subjects, Nikseresht et al. found a significantly reduced expression of plasmatic RANKL and OPG in patients with MASLD compared to controls [43]. A decreased expression of RANK was observed also in another study in subjects with MASLD [44]. Similarly, other research has described a negative association between OPG levels and MASLD [45,46]. Yang et al. reported that serum levels of OPG were even lower in subjects with steatohepatitis than in ones with simple steatosis [45]. We can speculate that a deficiency in OPG could potentially worsen liver inflammation because it usually plays an anti-inflammatory role. 

Following the previous studies, Zhang et al. confirmed the downregulation of hepatic OPG in murine models of liver steatosis and people with MASLD [47]. Surprisingly, they observed that OPG-knockout mice did not develop steatosis. Their hypothesis about this paradox is that OPG reduction in animals and people with MASLD could represent not the cause but rather a compensatory mechanism during liver injury development. 

All these data contrast with research conducted in patients with other liver diseases that have shown an increased expression of OPG [48,49]. For example, higher levels of OPG were found in patients with alcoholic liver disease (cirrhotic and non-cirrhotic) than in controls, while there was no difference in RANKL expression [50]. Similar results emerged from a study conducted in people with primary biliary cholangitis (PBC) [51]. Lleo et al. found that OPG and RANKL were widely expressed in PBC [52]. An improvement of OPG and RANKL serum levels in chronic liver disease could suggest a possible use for them as biomarkers of the activity of the disease, but more studies are needed to clarify if a clear correlation exists. 

The mentioned data do not allow us to make any conclusive statement about a possible correlation between serum levels of RANKL and OPG and the presence of MASLD. A possible explanation for these contrasting results could be derived from the first studies conducted on the bone. In fact, it has been established that RROa’s functions in bone remodelling are fundamentally mediated by paracrine stimulation [21]. We can hypothesise that the same happens for metabolic pathways, so RANKL and OPG blood levels could be profoundly different from their real concentration in the liver microenvironment. Moreover, multiple pieces of evidence show that OPG and RANKL are directly expressed on the surface of blood vessels [38], limiting the significance of their serum values.

As previously exposed, oestrogens play a key role in promoting bone formation while inhibiting osteoclastogenesis, through action on RROa. Interestingly, post-menopausal status represents a risk factor for both osteoporosis and MASLD [53]. This evidence suggests that oestrogen-mediated effects on RROa, which are well-known in bone metabolism, may be involved in liver physiopathology and in MASLD development.

The ubiquitous expression of sphingolipid-mediated pathways represents a further possible connection between liver and bone physiology and disease [54]. For example, it has been proved that sphingosine-1-phosphate, a sphingolipid mediator that is involved in osteoclastogenesis [55], is associated with in vitro hepatotoxicity and with liver inflammation after liver transplant in murine models [56]. At this moment, it is unknown if sphingolipids may be involved in mediating RROa effects on MASLD.

Regarding sclerostin, no data are yet available regarding the significance of this biomarker in relation to RROa in the context of MASLD or liver injury.

**Figure 2:**
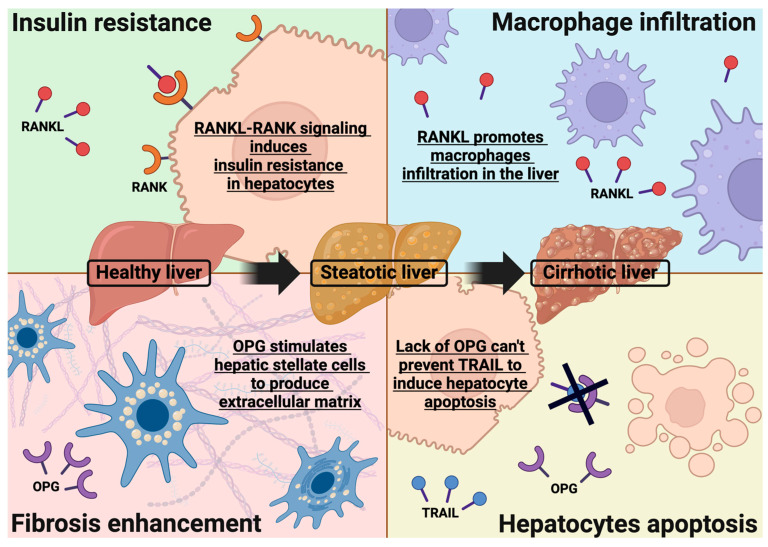
Possible involvement of the RANK–RANKL–OPG axis in MASLD development and progression. Current evidence highlights four main mechanisms of hepatic injury mediated by RROa: (1) RANKL–RANK signalling induces insulin resistance in hepatocytes, which is crucial for MASLD development; (2) RANKL promotes macrophage infiltration in the liver, and its levels gradually increase during disease progression; (3) OPG has a pro-fibrotic action stimulating HSCs to produce extracellular matrix; (4) OPG can block TRAIL, preventing it from inducing hepatocyte apoptosis, which is a progression factor for MASLD. (Abbreviation: RANKL: receptor activator of NF-κB ligand; RANK: receptor activator of NF-κB; OPG: osteoprotegerin; TRAIL: TNF-related apoptosis-inducing ligand).

Analysis of the specific biochemical interactions in the different biological functions where RANKL, RANK, and OPG are potentially involved can provide major information about their pathophysiological action in MASLD. As summarised in Figure 2, RROa can play a role in multiple steps of MASLD progression, which can be a direct or indirect source of liver injury.

### 5.1. Insulin Resistance

MASLD has a complex and multifactorial aetiology, but insulin resistance (IR), together with lipotoxicity, are cornerstones in the development and progression of the disease [4,57]. Kiechl et al., in the prospective population-based Bruneck Study, which involved 844 subjects, found that soluble RANKL levels represent an independent risk factor for the onset of type 2 diabetes mellitus (T2D) [58]. Furthermore, they used different murine models of obesity and metabolic syndrome to demonstrate that blocking the RANK–RANKL pathway in the liver significantly improved hepatic insulin sensitivity and normalised blood glucose levels.

IR also plays a major role in polycystic ovary syndrome (PCOS), and people with PCOS indeed have an increased risk of MASLD [59,60]. Recently, Lu et al. have shown a positive correlation between RANKL levels and the risk of suffering from MASLD in women with PCOS [61]. After adjusting for potential confounding factors, the subgroup analysis highlighted that the association was stronger in non-obese subjects than in the overweight and obese groups. This suggests that RANKL could be involved in IR and, therefore, in MASLD development independently of metabolic syndrome.

### 5.2. Macrophage Infiltration

Another pathophysiological mechanism underlying MASLD is the hepatic infiltration of macrophages [62]. This represents a critical element in promoting fat deposition and inflammation in the liver [62,63], determining disease progression from simple steatosis into steatohepatitis and, potentially, into cirrhosis. 

A study on murine models of MASLD has shown a direct correlation between the expression of RANKL and runt-related transcription factor 2 (Runx2) [64]. Runx2 represents a transcription factor initially identified as a fundamental element for osteoblastic differentiation and is involved in multiple biological pathways, like tumour proliferation, neoangiogenesis and inflammatory processes [65]. Runx2 also promotes hepatic infiltration of macrophages in steatosis [66], which is crucial for MASLD development and progression. Zhong et al. demonstrated that RANKL expression is directly correlated with the degree of macrophage infiltration of the liver, and its levels increase in parallel with the progression of the disease from simple steatosis into steatohepatitis [64]. This study strengthens the hypothesis of RANKL as a critical pro-inflammatory cytokine in promoting liver inflammation in MASLD.

### 5.3. Hepatic Fibrosis

Recently, some studies have highlighted OPG’s pro-fibrotic action [67], which could represent a transversal pathophysiological actor shared by any liver disease.

Resident immune cells of the liver play a major role in fibrosis development, particularly hepatic stellate cells (HSCs). HSCs are mesenchymal cells in a quiescent state located in the perisinusoidal space. They become active in case of liver damage, producing new extracellular matrix [68] and stimulating the expression of multiple growth factors and cytokine, including OPG [69]. One of the central cytokines that promote HSCs activation is TGF-β1, which stimulates OPG expression [70]. Therefore, it could create a vicious cycle where TGF-β1 promotes OPG expression, and OPG induces TGF-β1 production, determining a relevant autocrine/paracrine stimulation of HSCs’ fibrogenic activity.

Macrophage–HSC interaction is crucial for fibrosis progression [71]. As previously reported, RANKL can promote macrophage migration into the liver, while OPG can activate HSCs and trigger fibrotic processes [64,69]. Adhyatmika et al. proposed that OPG could be used as a marker of injury resolution in liver disease [69] because it promotes repairing mechanisms. Moreover, it could be proposed that beside its pro-fibrotic action, OPG carries out anti-inflammatory functions, blocking RANKL signalling.

### 5.4. Role of TNF-Related Apoptosis-Inducing Ligand (TRAIL)

OPG also acts as a decoy receptor for TNF-related apoptosis-inducing ligand (TRAIL) [72], a cytokine produced by monocyte–macrophage cells during the inflammatory response, which can promote cell apoptosis [73]. Human hepatocytes seem to be sensitive to the TRAIL-induced apoptosis pathway [74]. In TRAIL-knockout mice, Cartland et al. demonstrated an augmented risk of IR, T2D, and inflammation; thus, TRAIL-deleted murine models which undergo a high-fat diet developed a more severe form of MASLD [75]. On the contrary, Zheng et al. found that blocking the TRAIL pathway has a protective effect on the liver in a murine model of hepatitis [76]. TRAIL hyperexpression has also been previously observed in people with HBV infection [77].

Since hepatocyte apoptosis has been identified as an additional progression factor for MASLD [78], OPG could be involved in the pathogenesis due to its ability to block TRAIL-mediated effects. 

A reduced expression of OPG could promote MASLD progression due to the lack of inhibition of TRAIL and the consequent increase in hepatocyte apoptosis. This may suggest that TRAIL could represent a possible therapeutic target. However, it must be noted that TRAIL plays an important role in immunosurveillance because it can also induce apoptosis in neoplastic cells [79]. In this regard, multiple studies have shown a correlation between OPG upregulation and several tumours, such as prostate, breast and colorectal cancer [80,81,82,83,84]. Shi et al. have demonstrated the overexpression of OPG in pancreatic cancer, and they intriguingly highlighted that higher levels of OPG were associated with a higher incidence of new-onset diabetes [85]. Therefore, an excessive blockade of TRAIL by OPG can result in an increased risk of cancers, mainly HCC, in patients with MASLD. 

## 6. Targeting RROa with Medications: Denosumab

At present, the only drug with direct action on RROa is denosumab, a human monoclonal antibody that was approved in 2009 for the treatment of postmenopausal osteoporosis [86]. Fundamentally, denosumab mimics OPG’s physiological function because it binds RANKL with high affinity, preventing its interaction with RANK [87]. Denosumab produces a greater improvement in bone mineral density compared to bisphosphonates, which are still the cornerstone of pharmacological therapy for osteoporosis [88,89]. Furthermore, denosumab is a semestral subcutaneous administration, which could encourage the adherence to therapy, especially in patients with many comorbidities. A small retrospective study evaluated the efficacy and safety of denosumab administration in patients with chronic liver disease, showing an increased bone mineral density without occurrence of any severe adverse events [90].

The use of denosumab to treat MASLD or other liver diseases has never been tested, but speculation can be made due to an interesting case report: in 2016, Takeno et al. surprisingly reported an improvement in liver enzymes in a patient with non-alcoholic steatohepatitis (NASH) after denosumab administration [91]. More than a single case report is required to provide a clear therapeutic indication for denosumab in patients with MASLD; still, it indeed offers us a suggestive perspective for future research.

**Figure 3:**
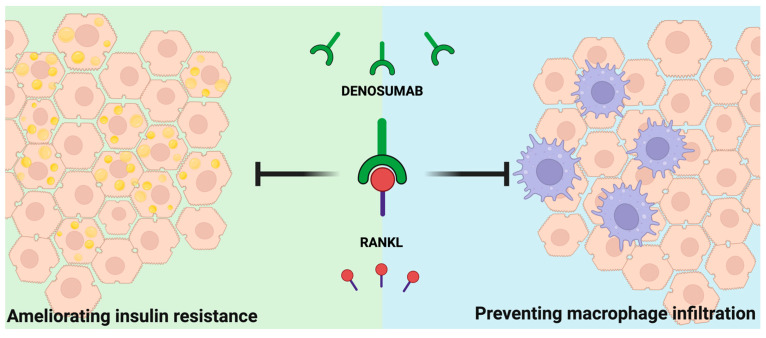
Hypothetical mechanism of action of denosumab in MASLD. RANKL inhibition by denosumab may ameliorate hepatic insulin resistance and prevent macrophage infiltration in the liver. (Abbreviation: RANKL: receptor activator of NF-κB ligand).

The possible effectiveness of denosumab treatment in MASLD could be hypothesised based on another work (Figure 3): compared to the placebo group, Weivoda et al. observed an improvement in glucagon-like peptide 1 (GLP-1) expression in subjects treated with denosumab [92]. GLP-1 belongs to the incretin family of hormones, and it improves insulin secretion. GLP-1 receptor agonists (GLP-1RA) represent a new class of antidiabetic drugs with impressive efficacy in multiple diseases, like obesity and heart failure. Furthermore, this study reported decreased HbA1c levels in the intervention group. Although the researchers did not focus on MASLD, their analysis highlights the pleiotropic effects of RANKL in metabolic pathways and again suggests that RROa could represent a novel pharmacological target in treating disorders across the metabolic syndrome spectrum.

A recent population-based study showed that denosumab treatment was associated with a reduced risk of developing diabetes over a mean follow-up period of 1.9 years. This suggests that this kind of anti-osteoporotic treatment could be considered in patients with osteoporosis and an increased risk of diabetes, like those presenting with metabolic syndrome or MASLD [93].

## 7. Anti-Osteoporotic Drugs for MASLD Treatment

Some studies have suggested that bisphosphonates, the most common anti-osteoporotic drugs, could be used as therapy for MASLD. Even if they do not target RROa, this evidence highlights once again the deep relationship between the bone and the liver.

Bisphosphonates exert their anti-resorptive effect by acting on mature osteoclasts through intracellular action, including the inhibition of the mevalonate pathway [84].

Hasuzawa et al. have tested the administration of clodronate—a non-nitrogen-containing bisphosphonate—in murine models of MASLD [94]. They found that clodronate induced a reduction in macrophage infiltration of the liver, an improvement in steatosis, and the prevention of fibrosis. The authors suggested that the effects of clodronate in MASLD models are due to its ability to block ATP secretion by hepatocytes [94], which has been related to liver inflammation and hepatic injury [95,96]. 

Two studies that evaluated the effects of zoledronic acid in murine models of MASLD have emerged with similar results, highlighting an improvement in hepatic fat content [97,98]. In contrast to clodronate, zoledronic acid seems to achieve an anti-steatotic effect through the inhibition of the mevalonic acid pathway [98].

Further data are needed about whether sclerostin inhibition could be an indirect strategy to target RRO to achieve certain metabolic effects. Romosozumab, a sclerostin inhibitor, is a human monoclonal antibody to treat severe osteoporosis [99]. However, concerns about possible increased cardiovascular risk during treatment will likely not make it the first choice to be tested in patients with MALSD, as opposed to denosumab.

## 8. RROa in Hepatocellular Carcinoma (HCC)

Hepatocellular carcinoma (HCC) is the most common primary liver cancer and represents the third cause of cancer-related death worldwide [100]. Although the incidence of HCC in MASLD is not as high as in HBV/HCV-driven cirrhosis, MASLD has a significantly higher prevalence and already represents the leading cause of HCC in the United States (US) [101]. Estes et al. suggested a possible increase of about 100% in MASLD-related HCC cases in Europe and the US by 2030 [102]. In patients with MASLD, HCC can develop even without underlying cirrhosis, differently from what happens in other chronic liver diseases [103]. The possible involvement of pathways mediated by RROa has been suggested in the development of MASLD-related HCC.

Song et al. have shown a hyperexpression and hyperactivation of RANKL–RANK signalling in HCC cells (HCCcs), which directly promotes migration and invasion of neoplastic cells in the liver parenchyma [104]. They also demonstrated that RANKL stimulation of RANK induces the expression of transcription factors linked with the epithelial to mesenchymal transition, a key step in HCC progression [104]. Activation of the NF-κB pathway by RANKL–RANK signalling was already associated with improvement in migration and tissue invasion by breast cancer cells [105]. A direct implication of RANK–RANKL in hepatic malignant transformation has already been proposed [106]. 

On the contrary, Sasaki et al. found no clear correlation between RANKL levels and HCCcs, but they showed that RANKL overexpression in patients with HCC positively correlates with the presence of bone metastases [107]. 

Some studies have indirectly confirmed the possible involvement of RANKL in HCC pathogenesis, pointing to a direct correlation between RANKL and the expression of the signal transducer and activator of transcription-6 (STAT6) [108]. STAT6 is a transcription factor involved in several cellular pathways and in cancer metabolism and progression [109,110]. It has been proposed that STAT6 may predict poor outcomes in patients with HCC [111]. 

If the upregulation of RANKL–RANK is involved in the development of HCC, it could be suggested that OPG, physiologically deputed to prevent RANK activation, may have a protective role. However, some evidence has shown a possible pathological role of high levels of serum OPG, even in HCC.

In fact, Zhang et al. have proved that higher OPG levels were associated with poorer survival rates in patients with HCC [112]. Previously, it was demonstrated that HCCcs constitutively express OPG and in hypoxic conditions, OPG expression can be significantly increased [113].

## 9. Conclusions

MASLD represents a challenging clinical issue with a massive social impact. Regrettably, its pathophysiological mechanisms are still largely unknown, limiting specific drug development. From this perspective, RROa is an intriguing target for researchers due to its wide range of functions and pleiotropic effects. Clearly, the emerging conflicting results make present knowledge insufficient to provide any underlying specific mechanism or biochemical marker of liver disease at the moment. 

To the present day, the main evidence has emerged from in vitro and murine model studies and this represents a major limitation in the direct application of these results in humans. On the contrary, the human studies available provide contrasting data and there is no unequivocal evidence regarding the specific pathways of RROa involvement in MASLD; available data tend to suggest it can play a role in MASLD development and progression, hitting several fronts simultaneously, besides the pivotal action in the pathophysiology of the disease, namely IR.

Further studies are needed to definitively clarify the role of RROa in human subjects with MASLD and the possible biochemical pathways involved. An understanding of these pathways will be essential to promoting research on new pharmacological targets.

Furthermore, it has been demonstrated that RROa is involved in other metabolic disorders, like T2D and obesity, that are deeply connected to MASLD. This suggests that RROa, born as a bone-exclusive pathway, can become a main target of research in metabolic disorders. In this scenario, even an anti-osteoporotic drug like denosumab could theoretically be tested to be repurposed from the bone to the liver.
